# Remote sensing of zooplankton swarms

**DOI:** 10.1038/s41598-018-37129-x

**Published:** 2019-01-24

**Authors:** Sünnje L. Basedow, David McKee, Ina Lefering, Astthor Gislason, Malin Daase, Emilia Trudnowska, Einar Skarstad Egeland, Marvin Choquet, Stig Falk-Petersen

**Affiliations:** 1 Department of Arctic and Marine Biology, UiT The Arctic University of Norway, Tromsø, Norway; 2 Physics Department, University of Strathclyde, Glasgow, United Kingdom; 3 Marine and Freshwater Research Institute, Reykjavik, Iceland; 4 Marine Ecology Department, Institute of Oceanology, Polish Academy of Sciences, Sopot, Poland; 5 Faculty of Biosciences and Aquaculture, Nord University, Bodø, Norway; 6 Akvaplan-niva AS, Tromsø, Norway

## Abstract

Zooplankton provide the key link between primary production and higher levels of the marine food web and they play an important role in mediating carbon sequestration in the ocean. All commercially harvested fish species depend on zooplankton populations. However, spatio-temporal distributions of zooplankton are notoriously difficult to quantify from ships. We know that zooplankton can form large aggregations that visibly change the color of the sea, but the scale and mechanisms producing these features are poorly known. Here we show that large surface patches (>1000 km^2^) of the red colored copepod *Calanus finmarchicus* can be identified from satellite observations of ocean color. Such observations provide the most comprehensive view of the distribution of a zooplankton species to date, and alter our understanding of the behavior of this key zooplankton species. Moreover, our findings suggest that high concentrations of astaxanthin-rich zooplankton can degrade the performance of standard blue-green reflectance ratio algorithms in operational use for retrieving chlorophyll concentrations from ocean color remote sensing.

## Introduction

Remote sensing of ocean color has led to major advances in understanding ocean-wide phytoplankton dynamics by providing much greater temporal and spatial coverage than is possible by ship-based sampling. Phytoplankton species rely on pigments for photosynthesis and other photophysiological processes. These different colored pigments affect the spectral characteristics of light redirected out of the sea and so becoming accessible to remote sensing detectors^1,2^. Surface-dwelling zooplankton, on the other hand, do not rely on capturing energy through pigments, and tend to be largely transparent to avoid the attention of visual predators. However, many crustaceans synthesize the red carotenoid astaxanthin from yellow and orange precursors in their diet and appear reddish^3^; this includes a few species that seasonally inhabit the surface ocean, such as copepods of the genus *Calanus*^4^. The widely distributed copepod *Calanus finmarchicus* (ca. 3 mm in size) is “often occurring in enormous shoals and thus sometimes giving the sea a conspicuous reddish hue”^5^, or “yellowish tint”^6^.

*C*. *finmarchicus* is a key species in the North Atlantic ecosystem, frequently constituting > 50% of mesozooplankton biomass^7^. Due to its large biomass and high lipid content this species is the principal prey for some of the world’s largest fish stocks, such as larvae of Northeast Arctic cod (*Gadus morhua*) and adult Norwegian spring spawning herring (*Clupea harengus*)^8^. It is also important for the transfer of energy to most marine animals at higher trophic levels throughout the ecosystem^9^. *C*. *finmarchicus* is also commercially exploited for the rich omega-3 fatty acid content of its lipids^10^. Intense UV-radiation at the sea surface can degrade the copepods’ lipids through peroxidation^11^, but nonetheless during spring and summer, species of the genus *Calanus* have repeatedly been observed right at the sea surface^12–17^. It has been suggested that one reason copepods synthesize astaxanthin is that it protects against oxidative damage of lipids by scavenging free radicals^3,11^. Due to its red astaxanthin pigmentation, and its importance as food for commercially important fish species, *C*. *finmarchicus* has been given the local common names ‘raudåte’ (Norwegian) and ‘rauðáta’ (Icelandic), meaning ‘red feed’.

Aggregative behavior is a universal feature in a wide range of animals^18,19^, and the majority of marine animals aggregate to some extent^20–24^. This includes zooplankton organisms that are mostly smaller than 4 mm and possess limited mobility^25^. However, mechanisms for spatial aggregations of zooplankton are still poorly understood and remain a central issue in marine ecology^26–28^. Progress is hindered by the difficulty of mapping zooplankton abundance and distribution from ships. Thus, although several sporadic observations indicate surface aggregations of *Calanus* spp. during daylight^12–17^, the frequency of occurrence and the extent of these zooplankton surface aggregations are not known. This impedes our ability to identify behavioral and physical drivers for zooplankton aggregations, and in turn inhibits our understanding and modelling of the consequences for the ecosystem.

Here we test the hypothesis that the presence of high concentrations of the pigment astaxanthin will enable discrimination of zooplankton surface swarms from phytoplankton blooms in ocean color remote sensing signals. By pairing satellite images with high-resolution *in situ* zooplankton data, and with detailed measurements of optical properties of seawater and of *Calanus* spp. at oceanographic stations, we aim to establish the potential for remote sensing of this copepod.

## Results

### Large-scale zooplankton surface swarms

**Figure 1:**
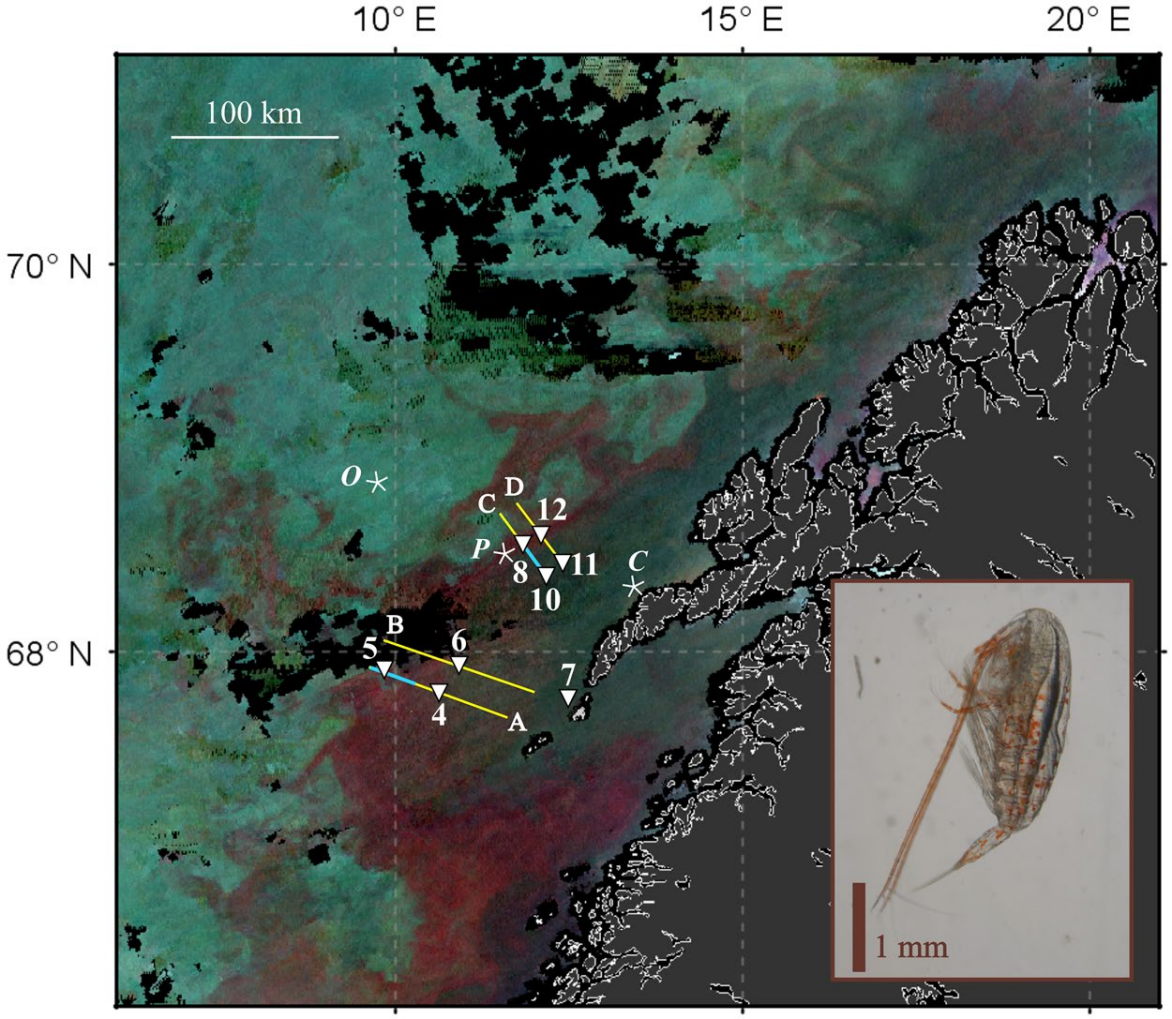
Weekly composite of VIIRS RGB images obtained during 27 April – 3 May 2017, showing large areas of red pixels. *In situ* sampling was performed in the study area off northern Norway between 28 April to 4 May 2017, using optical plankton counters towed along transects A to D (laser optical plankton counter – yellow lines, video plankton recorder – blue lines), and at eight stations (white triangles). Locations from which pixels were extracted for comparing Remote Sensing Reflectance (Fig. 3) are marked with white asterisks; *O* – open ocean, *P* – inside patch, *C* – coastal. The copepod *Calanus finmarchicus* (developmental stage CV) with red astaxanthin pigmentation in the antenna and across its body is depicted in the inset. Fully processed data for the satellite image were provided by NEODAAS.

**Figure 2:**
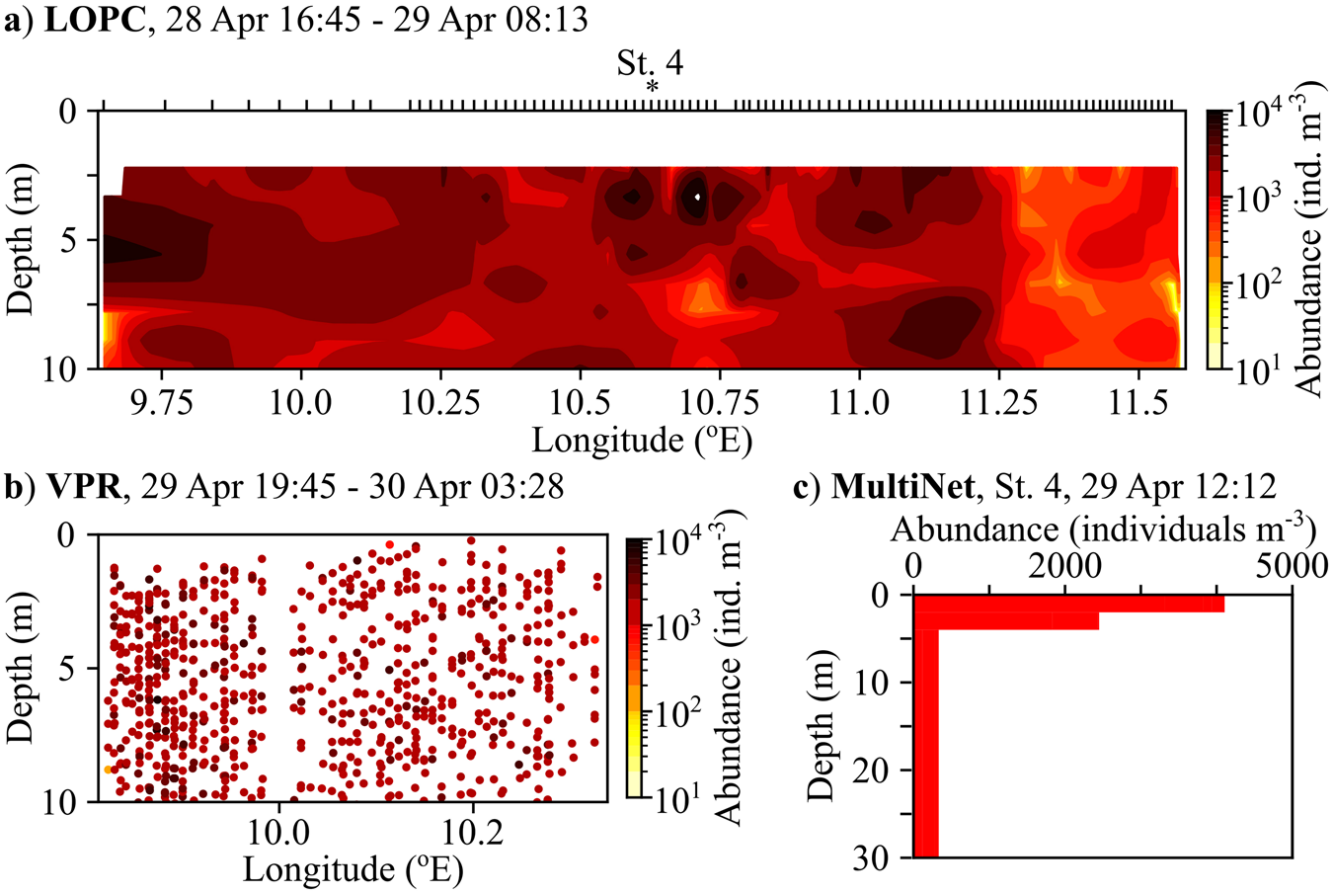
Abundance and distribution of older developmental stages of *Calanus* spp. (copepodites IV and older) in the upper 10–30 m along transect A as observed by (**a**) a laser optical plankton counter, (**b**) a digital auto video plankton recorder, and (**c**) a MultiNet at station 4. Ticks along the top axis of panel a indicate start points for vertical sampling profiles.

During an oceanographic cruise off the coast of Northern Norway in May 2017 large areas of red pixels were visible in enhanced Red-Green-Blue (RGB) images obtained from the VIIRS (Visible Infrared Imaging Radiometer Suite) satellite (Fig. 1). Similar features were observed in concurrent images from other ocean color satellite systems (not shown). The main patch stretched from the coast across the shelf and northward along the shelf break. It covered an area of several thousand km^2^, with mesoscale structures visible along the shelf break. At the same time, a suite of *in situ* instruments detected very high abundances of *Calanus* spp. in the surface layer (Fig. 2, Supplementary Figs 1–3), at locations matching the red pixels. Most of the observed copepods were highly pigmented, and many contained pigments not only in the lipid sac and antenna, but also in the carapace (Fig. 1, inset). Genetic analyses confirmed that *C*. *finmarchicus* was the dominant *Calanus* species in the patch, with a few individuals of the closely related *C*. *glacialis* identified at stations 6 (2 ind.) and 7 (1 ind.). Smaller-scale features within the large red-pixel patch were resolved by *in situ* optical plankton recorders (laser optical plankton counter and video plankton recorder). These instruments detected horizontal and vertical structures within the patch, and both sensors recorded abundances of >1,000 *Calanus* spp. m^−3^ in the upper 10 m (Fig. 2, Supplementary Figs 1 and 2). Abundance estimates based on net samples (180 µm mesh) were of the same order of magnitude, and at 7 out of 9 stations the highest abundance of pigmented copepods (copepodite stage IV and older) was observed in the upper 5 m (Fig. 2, Supplementary Fig. 3).

### Remote sensing reflectance and absorption of the copepod’s pigments

**Figure 3:**
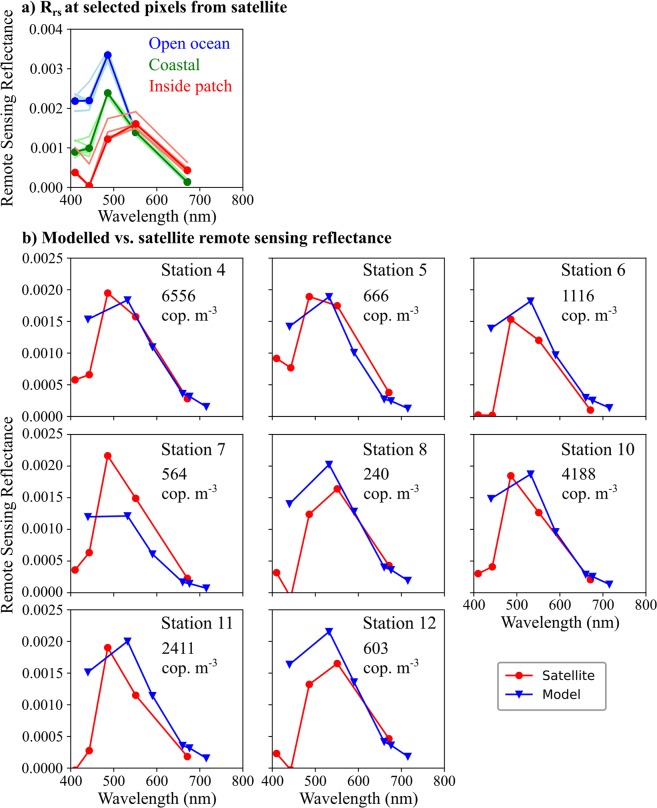
Remote sensing reflectance. (**a**) At three selected locations (nine pixels each, thin lines): in the open ocean (blue), inside the red pixel patch (red), and in a coastal area (green). Thick line: mean over the nine pixels. (**b**) At 8 stations in the study area. Red lines: VIIRS satellite data, blue lines: radiative transfer model populated with inherent optical properties measured at the stations. The *Calanus* spp. abundance in the upper 5 m as observed by MultiNet is indicated for each station.

**Figure 4:**
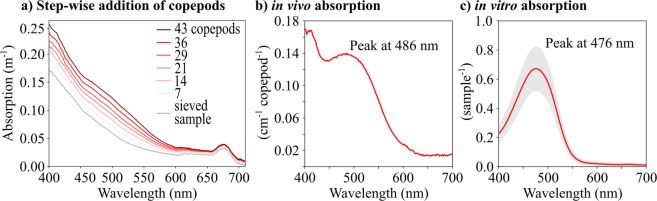
Light absorption of *Calanus* pigments. (**a**) Absorption with increasing number of copepods during serial addition to a point source integrating cavity absorption meter (PSICAM) (**b**) *In vivo* absorption measured in the PSICAM. (**c**) *in vitro* absorption measured spectrophotometrically from pigments extracted in acetone. Thick line: mean over all 21 samples, gray area: 95% confidence interval.

Comparison of satellite remote sensing reflectance (R_rs_) spectra from inside the patch, in the open ocean, and in a coastal area (Fig. 3a) showed blue reflectance decreasing from open ocean to coastal waters as expected, and then decreasing further inside the patch. Radiative transfer modelling using *in situ* measurements of inherent optical properties from inside the patch consistently overestimated R_rs_ at wavelengths <550 nm compared with data from remote sensing (Fig. 3b). These observations led us to perform a serial addition experiment, where the absorption of copepods was measured in a point source integrated cavity absorption meter (PSICAM), which has previously been shown to measure absorption without errors due to particle scattering^29^. *C*. *finmarchicus* were found to absorb strongly in the blue-green, and absorption increased linearly with copepod numbers (Fig. 4a). The absorption of *C*. *finmarchicus* extended across the blue-green spectrum with a peak at 486 nm, where the discrepancy between modeled and measured R_rs_ was detected (Fig. 4b). The absorption of astaxanthin extracted from copepods in the laboratory peaked at 476 nm in acetone (Fig. 4c), confirming the role of the pigment in driving *Calanus* sp. absorption.

### *In situ* phytoplankton and satellite chlorophyll

**Figure 5:**
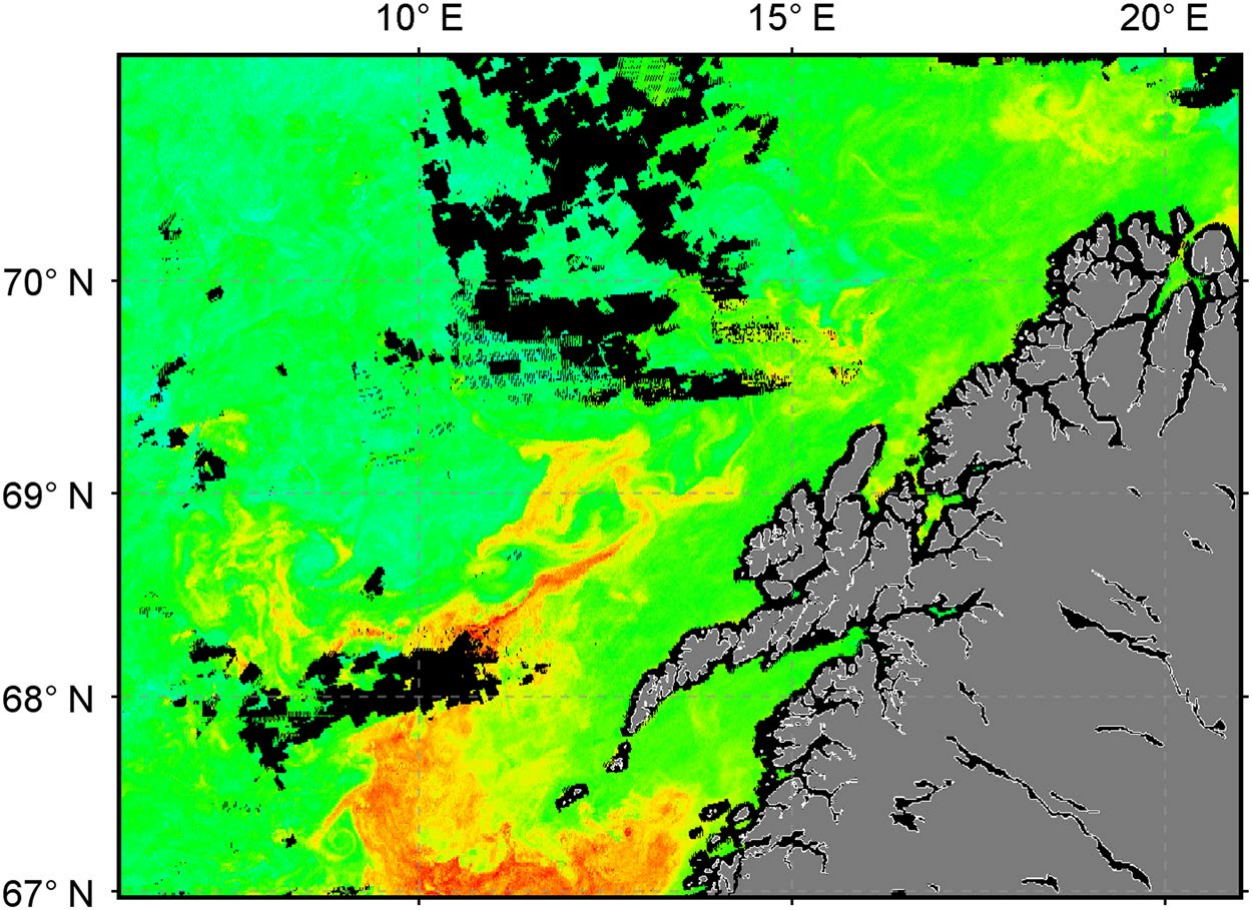
Weekly composite of VIIRS chlorophyll *a* during 27 April – 3 May 2017. Fully processed data for the satellite image were provided by NEODAAS.

**Figure 6:**
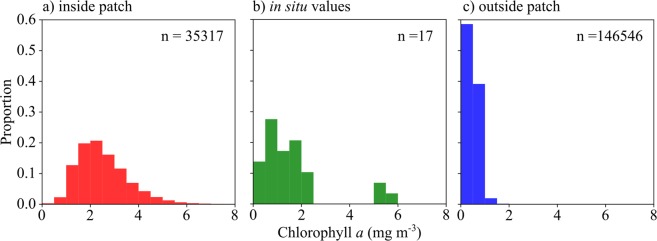
Chlorophyll *a* concentration in the study area. (**a**) Measured by satellite inside the patch, defined as R_rs_ > 4 × 10^4^ sr^−1^ (**b**) measured fluorometrically from filtered water samples (**c**) measured by satellite outside the patch, defined as R_rs_ < 2 × 10^4^ sr^−1^.

*In situ* measurements of phytoplankton were relatively low, both in cell numbers (<50 × 10^3^ cells m^−3^) and biomass (mostly < 2 mg chlorophyll *a* m^−3^). The spatial distribution of satellite-recorded chlorophyll *a* (Fig. 5) was essentially the same as that of the red pixels (Fig. 1) and both *in situ* and VIIRS remotely sensed chlorophyll concentrations inside the patch showed a similarly broad range of values up to approximately 6 mg m^−3^ (Fig. 6). However, the distribution of in patch VIIRS chlorophyll *a* data (Fig. 6a) was skewed to higher values, with very few data points < 1 mg m^−3^. In contrast, the *in situ* chlorophyll *a* data from the upper 5 m had a greater proportion of values < 1 mg m^−3^ (Fig. 6b) that was broadly consistent with the VIIRS data from outside the patch (Fig. 6c). It thus appears that there may be a tendency for the standard blue-green reflectance ratio algorithm to return overestimates of chlorophyll *a* for waters with high abundances of astaxanthin-rich zooplankton.

## Discussion

The results corroborate our hypothesis that high abundances of astaxanthin-rich zooplankton in surface waters can influence bulk optical properties of these waters, and can be detected by ocean color remote sensing. To the best of our knowledge, this is the first time a larger, metazoan zooplankton species has been observed from space. The recording of super-swarms of *Calanus* spp. copepods at the sea surface alters our understanding of the behavior of this key zooplankton species in spring. Herbivorous copepods tend to display a flexible, unsynchronized, vertical migration behavior to balance food availability against predation pressure^30,31^. However, our observations of large-scale surface aggregations during daylight hours indicate that *Calanus* spp. at higher latitudes might maximize fitness by remaining in surface waters during the short productive season^32,33^. Note that our study region experiences 24 hours daylight from approximately mid-May to mid-July. Across the North Atlantic and the North Pacific, species of the genus *Calanus* have been observed in dense aggregations at the sea surface^12–17,34^. The new observations show that historical local sightings may be features of much larger spatial extent, involving vast numbers of animals of the utmost importance to fisheries and the broader marine food web.

The observed discrepancy between modeled and measured remote sensing reflectance (R_rs_) at blue wavelengths (Fig. 3b) can be attributed to copepod absorption as revealed by the PSICAM measurements (Fig. 4a,b). These *in vivo Calanus* sp. absorption data are broadly consistent with the *in vitro* absorption of the copepods’ astaxanthin pigment in acetone (Fig. 4c), as well as with previous measurements of astaxanthin absorption in deep sea zooplankton^35^. Other astaxanthin-rich zooplankton such as krill likely absorb at similar wavelengths^3^. R_rs_ was modeled using measured *in situ* IOPs, with an approach that has previously yielded good agreement with *in situ* radiometry^36^. However, in this study we attribute the discrepancy in the blue-green to the sample volumes of the *in situ* IOP sensors being too small (maximal 30 mL) to resolve adequately the optical properties of *C*. *finmarchicus* and other astaxanthin-rich zooplankton, even at the high abundances observed here. Although the recorded abundances of *Calanus* spp. are comparatively high, quantifying abundance in the surface layer around large ships is challenging due to mixing by thrusters and other local disturbances. We thus cannot rule out that the copepods were concentrated in a very thin layer at the sea surface prior to our obtrusive sampling. There may also be effects associated with evasion response behaviors by larger astaxanthin-rich zooplankton, reducing the signals recorded by *in situ* sensors. Most krill species are strong swimmers and able to evade even towed sampling systems during daylight hours^37^. Astaxanthin-rich zooplankton optical signals (primarily absorption) were thus underrepresented in the radiative transfer model. In contrast, the satellite sensor does not disturb the zooplankton population and each satellite pixel integrates over much larger spatial scales (>100 m) with the result that the observed, high abundances of *C*. *finmarchicus*, and potentially larger, under-sampled zooplankton species, appear to have measurably influenced satellite R_rs_. This highlights a general need to consider the impact of large (>1 mm), colored particles, such as but not limited to *C*. *finmarchicus*, on the propagation of light in the ocean and hence on R_rs_ signals. There is also a corresponding need to develop new techniques to adequately resolve IOPs of waters containing these particles.

The study area is highly advective, with eddies developing between the Norwegian Coastal Current that flows northeastward along the shore, and the saltier Norwegian Atlantic Current that flows in the same direction but further offshelf^38^. The main patch of *Calanus* spp. followed the shelf edge in a relatively narrow band from station 5 to station 8 and 12 (Fig. 1). Strong spatio-temporal variability limited our ability to perform direct comparisons between individual *in situ* and remotely sensed observations. At stations 5, 8 and 12, for example, relatively low *Calanus* spp. abundances were observed in net samples, but satellite R_rs_ was nevertheless substantially reduced in the blue-green (Fig. 3b), and the optical zooplankton sensors LOPC and VPR registered high abundances in that area between 28 and 30 April (Fig. 1, Supplementary Figs 1, 2). The most likely explanation is that the satellite captured a patch during the early days in the composite image (27 to 29 April), and that the patch had moved slightly when we took net samples (30 April to 3 May, Table 1) so that we were sampling in areas classified as “open ocean” according to the satellite image.

**Table 1:** Selected measurements at stations in the study area.

Station	Latitude (°N)	Longitude (°E)	Date	Time (UTC)	Gear
4	67.78	10.63	29 Apr	10:56	CTD
	67.78	10.62		12:12	MultiNet
	67.79	10.59		13:59	Hyperspectral sensor
	67.80	10.60		14:36	Surface water for IOPs
5	67.91	09.84	30 Apr	06:26	CTD
	67.90	09.84		07:25	MultiNet
	67.90	09.85		08:46	Hyperspectral sensor
	67.91	09.86		09:11	Surface water for IOPs
6	67.93	10.92		15:11	Hyperspectral sensor
	67.93	10.92		15:41	Surface water for IOPs
	67.93	10.93		17:26	CTD
	67.93	10.94		18:00	MultiNet
7	67.75	12.51	01 May	00:49	MultiNet
	67.75	12.51		01:05	CTD
	67.75	12.51		03:49	Hyperspectral sensor
	67.75	12.52		04:26	Surface water for IOPs
8	68.58	11.84		14:53	Hyperspectral sensor
	68.58	11.84		ca. 15:10	Surface water for IOPs
	68.58	11.84		15:22	CTD
	68.58	11.77		18:08	MultiNet
10	68.42	12.16	02 May	13:05	Hyperspectral sensor
	68.41	12.18		13:38	Surface water for IOPs
	68.44	12.18		15:27	CTD
	68.40	12.19		15:58	MultiNet
11	68.42	12.42	03 May	03:33	CTD
	68.47	12.43		06:06	MultiNet
	68.47	12.45		06:34	Hyperspectral sensor
	68.47	12.46		07:04	Surface water for IOPs
12	68.63	12.11		08:36	CTD
	68.63	12.12		08:58	Hyperspectral sensor
	68.63	12.13		09:25	Surface water for IOPs
	68.63	12.13		09:58	MultiNet

The discrepancy between satellite and modeled R_rs_ cannot be attributed to phytoplankton. Algal optical properties were well accounted for by the *in situ* IOP measurements, and were therefore included in the modeled R_rs_ spectra (Fig. 3b). Evidence to support this is found in the PSICAM data, where the absorption peak around 670 nm (Fig. 4a) is due to chlorophyll *a*. Very importantly, by influencing absorption and R_rs_ in the blue-green, high abundances of *Calanus* spp. and other astaxanthin-rich zooplankton could potentially affect the performance of standard blue-green reflectance algorithms, such as those used to estimate chlorophyll *a* concentrations by ocean color remote sensing (e.g.^39,40^). The distribution of VIIRS chlorophyll *a* (Fig. 5) shows strong spatial correlation with red pixels in the RGB image (Fig. 1). Due to the relatively low number of *in situ* measurements of chlorophyll *a*, and limited spatio-temporal match-ups, our data in this respect are not conclusive. However, the fact that *Calanus* spp. absorbed light strongly across the blue-green spectrum, and were present in high abundances at the surface in these waters, provides a plausible mechanism for influencing the bulk IOPs of the water, and hence the R_rs_ signals in the blue-green. It therefore seems likely that standard blue-green ratio algorithms to measure satellite chlorophyll *a* might be generally impacted in areas with high surface abundances of *Calanus finmarchicus* or other astaxantin-rich zooplankton.

On the positive side, the observed dependence of absorption on copepod numbers strongly suggests potential for development of new algorithms to estimate the abundance of a key species in the North Atlantic marine ecosystem. Quantitative remote sensing of surface abundances of the copepod *C*. *finmarchicus* might be possible in the near future. However, developing a quantitative algorithm for estimating total abundance of *Calanus* spp. is challenging because of the diel and seasonal vertical migrations performed by these copepods. Empirical relationships between surface abundance and abundance in deeper layers could potentially be established based on the vast amount of literature describing these migrations. Nevertheless, the benefits of remotely detecting surface *C*. *finmarchicus* abundance are substantial, and include increased understanding of zooplankton swarming behavior, better informed sustainable management of this harvested key species, and the possibility to track the species’ observed range expansion with climate change^41^. Here we report on the occurrence of red pixels in VIIRS RGB images associated with high abundances of *C*. *finmarchicus* in the Norwegian Sea during one week in early May 2017. We subsequently observed similar red pixel features in RGB images obtained on cloud-free days later in the season. Furthermore, the harvesting company Calanus AS reports surface swarms to occur regularly in this region between May and July (Kurt Tande, pers. comm.). This indicates that large-scale surface aggregations of *Calanus* sp. are a recurrent feature. We expect similar large-scale surface aggregations to occur in other higher latitude systems, where the productive season is short, and where local surface aggregations have been observed^12–17,34^. Remote sensing of *Calanus* spp might be possible in these systems as well, but the universality of the observed features remains to be established.

## Material and Methods

### Field sampling

*In situ* data were collected during a cruise on R/V “Helmer Hanssen” from April 28 to May 4, 2017 off the coast of northern Norway. Semi-automatic optical sampling of zooplankton was performed along four transects (Fig. 1). Irradiance, inherent optical parameters (IOPs), chlorophyll *a* concentration, phytoplankton abundance, and the abundance, vertical distribution, genetic species identity and pigment absorption of *Calanus* were measured at 8 stations within the study area (Table 1).

### Satellite images

Real-time support from NEODAAS (NERC Earth Observation Data Acquisition and Analysis Service) provided RGB images and NetCDF data files from daily (cloud-free) VIIRS satellite overpasses, which were used to map water masses with distinct spectral signatures and provided a first indication on the location of red, i.e. potentially *Calanus*-rich, patches. NEODAAS also provided weekly composite images such as that shown in Fig. 1 (RGB) and 5 (chlorophyll *a*), from 27 April to 3 May 2017. April 27–29 contributed most to the weekly average image, due to relatively high percentage of cloud cover on the later days. To generate the RGB image, wavebands 551 nm, 486 nm and 443 nm were used, and each color was scaled to include 95% of all pixels. A gamma correction of 0.9 was applied to the blue channel to improve contrast. To illustrate the differences in spectral R_rs_, three areas (three times three pixels from each area) with three different colors were selected randomly from the weekly composites, representing an open ocean site (blue), a *Calanus* spp. patch (red) and a coastal site (green) (Fig. 3a). Satellite image pixels were determined to be inside the patch if red reflectance, R_rs_(671 nm), was greater than 0.004 steradian^−1^ (sr), while those pixels with R_rs_(671 nm) <=0.002 sr^−1^ were determined to be outside the patch, with intermediate values of red reflectance forming an unattributed boundary region. Additionally, VIIRS R_rs_ spectra were extracted from fully processed NetCDF files provided by NEODAAS. R_rs_ spectra measured at the pixel closest to each sampling location were selected from the weekly average (for maximum number of cloud-free pixels) image and used for comparison against modeled R_rs_ spectra (Fig. 3b). These R_rs_ spectra are for illustrative purposes only as the temporal mis-match for any given match-up with the weekly composite image may be significant.

### Zooplankton data

Along the transects (Fig. 1, yellow lines) optical zooplankton data were obtained with high spatial resolution using a Moving Vessel Profiler (MVP, ODIM-Brooke Oceans Rolls Royce Canada, Ltd.) equipped with a Laser Optical Plankton Counter (LOPC, ODIM-Brooke Oceans Rolls Royce Canada, Ltd.). The LOPC was towed behind the ship at 6 to 7 knots, mounted in a “fish” profiling between surface waters down to 10 m above the seabed at 3.5–4 m s^−1^ vertical speed. The LOPC counts and measures plankton and particles at 2 Hz in the size range from 0.1 to 35 mm, which pass through its sampling tunnel. Data were quality-controlled ensuring that sample abundance was sufficiently low to avoid erroneous counts. Particles with the characteristics (transparency and size) of older stages of *Calanus* spp. (copepodites stages IV and older) were extracted from the data as described in^42^. Figures of the spatial distribution were prepared by interpolating between the vertical profiles across the transects, using the function griddata in the python library scipy.interpolate (www.scipy.org version 0.18.1).

After completion of the semi-automatic MVP sampling, sections of two transects (Fig. 1, blue lines) were revisited with a digital auto Video Plankton Recorder (VPR, Seascan Inc.). The VPR is equipped with a camera that takes colour images at a rate of up to 15 Hz. From the images the abundance and distribution of *Calanus* spp. (copepodite stages IV and older) was estimated as in^17^. The VPR was towed along transects in a saw-tooth trajectory between the surface and ~50 m depth. Towing speed was 2.5–3 knots, and the vertical speed of the VPR was ~0.5–1 m s^−1^.

The abundance of *Calanus* spp. at each station was quantified based on vertically stratified net samples collected by a MultiNet Midi (0.5 m s^−1^ hauling speed, 180 µm mesh size, 0.25 m^2^ mouth opening, Hydro-Bios, Germany). Samples were preserved in a solution of 80% seawater and 20% fixation agent (75% formaldehyde buffered with hexamine, 25% anti-bactericide propane-1,2-diol), resulting in a final formaldehyde concentration of 4%. Sub-samples from the depth layers 30 to 5 or 4 m, 5 or 4 to 2 m, and 2 m to surface were enumerated under a stereo microscope. At least 450 zooplankton individuals and about 100 *Calanus* were identified from each sample. Abundance was calculated based on the filtered volume obtained from the MultiNet flow meter.

Live *Calanus* copepodite stages CIV, CV and adult females were collected from the upper 15 m using a Bongo net (mouth opening 0.28 m^2^, mesh size 200 and 500 µm) that was towed for 10 minutes at 2 knots, at all stations except station 12. For pigment analyses triplicates of 30 to 39 *Calanus* spp. CV were frozen at −80 °C until analysis ashore. Copepods from one triplicate were photographed under the microscope prior to freezing. On land, frozen copepods were mixed with acetone under nitrogen and stirred until the biological material was colorless. The filtered acetone extract was evaporated under reduced pressure to dryness, re-dissolved in 0.5 ml acetone and light absorption measured by a UV/visible light spectrophotometer (Cary 100 UV-Vis, Agilent), Fig. 4c. From the frozen Bongo net samples, triplicates of individual *Calanus* spp. (CV and females) were selected for molecular analyses. Copepods were identified to species level using six molecular InDel markers and following the procedure described in^43^.

### Phytoplankton data

Chlorophyll *a* concentrations were determined fluorometrically (10-AU fluorimeter, Turner designs, USA) from pigment extracts in methanol obtained from filtered water samples (triplicates of 50 to 150 mL, GF/F filters, Whatman Inc., USA) from 0, 5, 10, 25, 50 and 100 m depth at all stations. Water samples were collected using Niskin bottles, filtered on board, and kept frozen at −80 °C before analysis ashore immediately after the cruise. Water samples for phytoplankton enumeration were collected from 0, 5 and 10 m at stations 11 and 12. They were fixed in a solution of formaldehyde in seawater with a final concentration of 4% formaldehyde. Ashore, samples were analyzed under the microscope after settling for 24 hours in Utermöhl chambers^44^.

### Bio-optics and radiative transfer modelling

Spectral absorption, attenuation and backscattering coefficients (inherent optical properties, IOPs) were determined from surface water samples collected with a bucket. Spectral absorption and attenuation coefficients were determined at nine different wavebands centered on 440, 532, 590, 660, 676, 715, 750, 820 and 870 nm using an *in situ* spectrophotometer (ac-9, WET Labs Inc., USA) equipped with 10 cm flow tubes. The ac-9 was calibrated on board at the start and end of the cruise using purified water. At each station, at least 8 L surface water were carefully pumped through the instrument avoiding accumulation of bubbles inside the cuvettes (sample volume ca. 30 mL). Raw spectra were obtained from average values of all spectra for each surface water sample. Absorption and attenuation were corrected for changes in pure water absorption due to temperature and salinity effects using the coefficients by Röttgers *et al*.^45^. Absorption spectra were further corrected for scattering errors by applying the proportional correction^46^ using 870 nm as reference wavelength.

Spectral backscattering coefficients were calculated based on measurements of the volume scattering function at an effective scattering angle of 124° (sample volume ca. 1 mL). Data were recorded inside a black bucket using a BB9 (WET Labs Inc., USA) with a total of 18 kbyte data collected over a period of several minutes. Averaged volume scattering at 124° function data were converted to particulate backscattering coefficient as described in the BB9 manual using χ = 0.9^44^. Spectral backscattering coefficients were determined at 412, 440, 488, 510, 532, 595, 650, 676 and 715 nm, which were subsequently interpolated to the ac-9 wavelengths.

Underwater and water-leaving light fields were simulated using EcoLight (version 5.0, Sequoia Scientific Inc., USA), a well-established radiative transfer model. The model was populated with measured IOPs and set up to generate Fournier-Forand scattering phase functions from derived particulate backscattering ratios^47,48^. Measured median above surface irradiance and estimated cloud cover percentage were used to parameterise incoming solar irradiation. Modelling of inelastic scattering was restricted to Raman scattering by water. Output was computed at IOP wavelengths to avoid artefacts due to inter- or extrapolation of the data.

The absorption of live copepods was measured in triplicate in the PSICAM between 350 and 800 nm using purified water as reference. A serial addition experiment was performed by sequentially adding seven to eight live *Calanus* spp. to sea water (Fig. 4a), which had been sieved through a 180 µm mesh to remove other large particles. The PSICAM has been shown to be virtually unaffected by scattering errors^29^ and is ideally suited to measure absorption of samples containing particulate matter such as *Calanus* spp. copepods. The PSICAM was calibrated multiple times per day using a Nigrosine solution^29,49^, and corresponding Nigrosine absorption spectra were measured using a long pathlength system. PSICAM absorption spectra were corrected for temperature and salinity effects using instrument specific correction factors^50^.

## Supplementary information

Supplementary Figures S1 to S3

## Data Availability

Remote sensing data from VIIRS were provided by the NERC Earth Observation Data Acquisition and Analysis Service. All other data that support the findings of this study are available from the corresponding author upon reasonable request.
