# Supplementary material for: Remote sensing of zooplankton swarms
Source: Sci Rep. 2019 Jan 24;9:686. doi: 10.1038/s41598-018-37129-x (PMC6346024; doi:10.1038/s41598-018-37129-x)
Supplement: Supplementary file 1 — Supplementary Figures S1 to S3 [file 41598_2018_37129_MOESM1_ESM.pdf]

Supplementary Material

Remote Sensing of Zooplankton Swarms

Sünnje L. Basedow*[1], David McKee[2], Ina Lefering[2], Astthor Gislason[3], Malin Daase[1], Emilia Trudnowska[4], Einar Skarstad Egeland[5], Marvin Choquet[5], Stig Falk-Petersen[6,1]

*** Correspondence:** Corresponding Author: sunnje.basedow@uit.no

3 Figures.

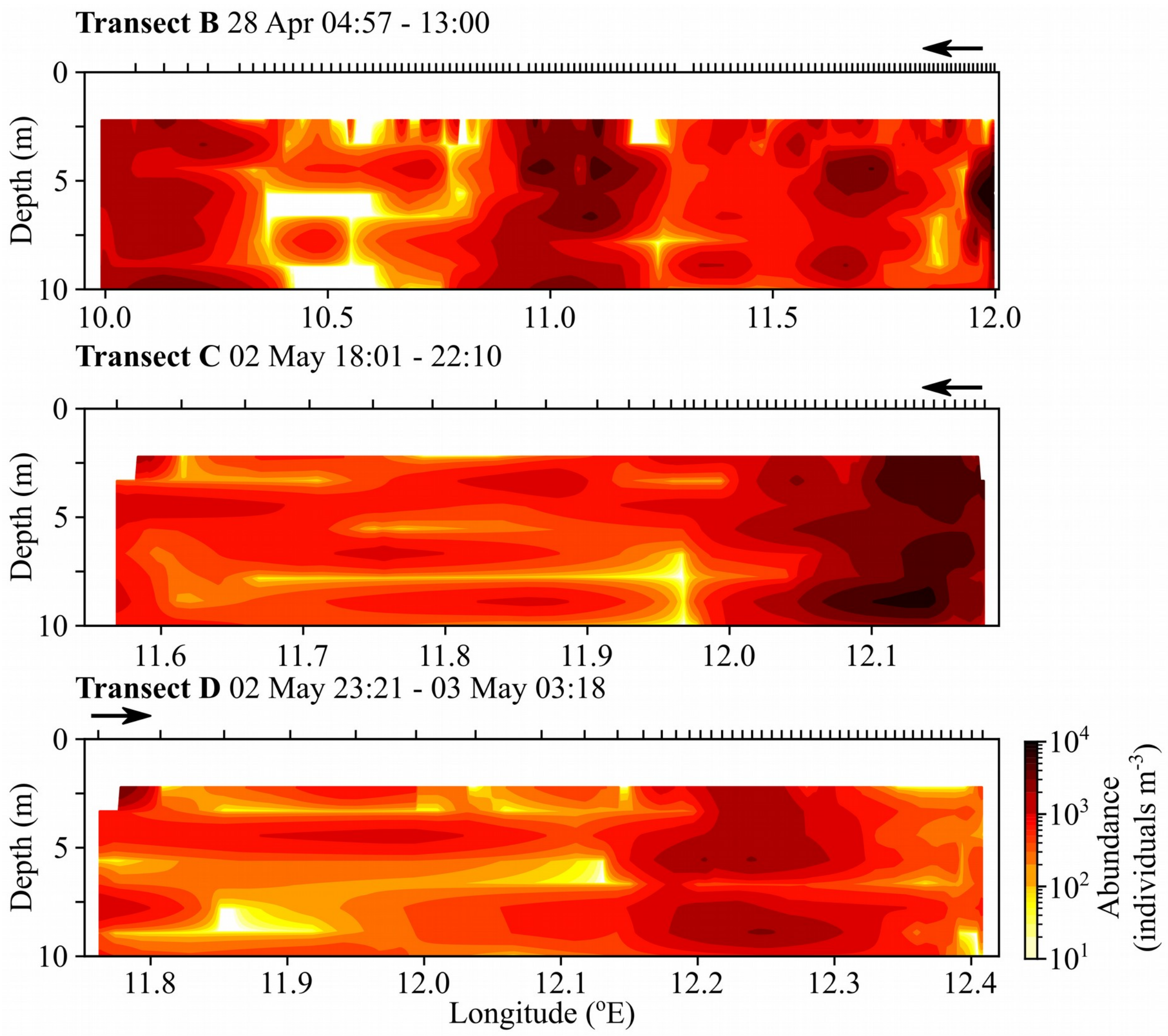

Fig. S1 Abundance and distribution of older developmental stages of *Calanus* spp. (copepodites IV and older) as observed by a laser optical plankton counter in the upper 10-30 m along three transects off the coast of northern Norway. Ticks along the top axis indicate start points for vertical sampling profiles, and the arrows indicate from which end of transect sampling started. See Fig. 1 for the location of transects.

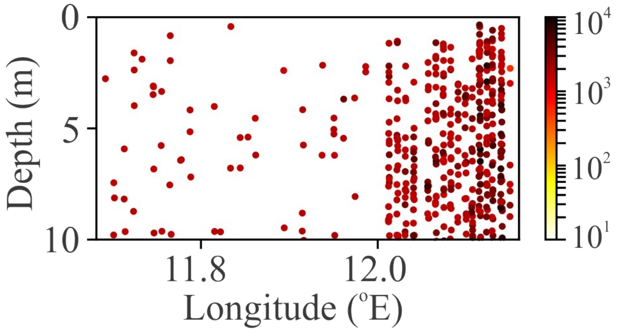

Fig S2. Abundance and distribution of older developmental stages of *Calanus* spp. (copepodites IV and older) as observed by a video plankton recorder in the upper 10 meters along transect C off the coast of northern Norway in May 2017. See Fig. 1 for the location of the transect.

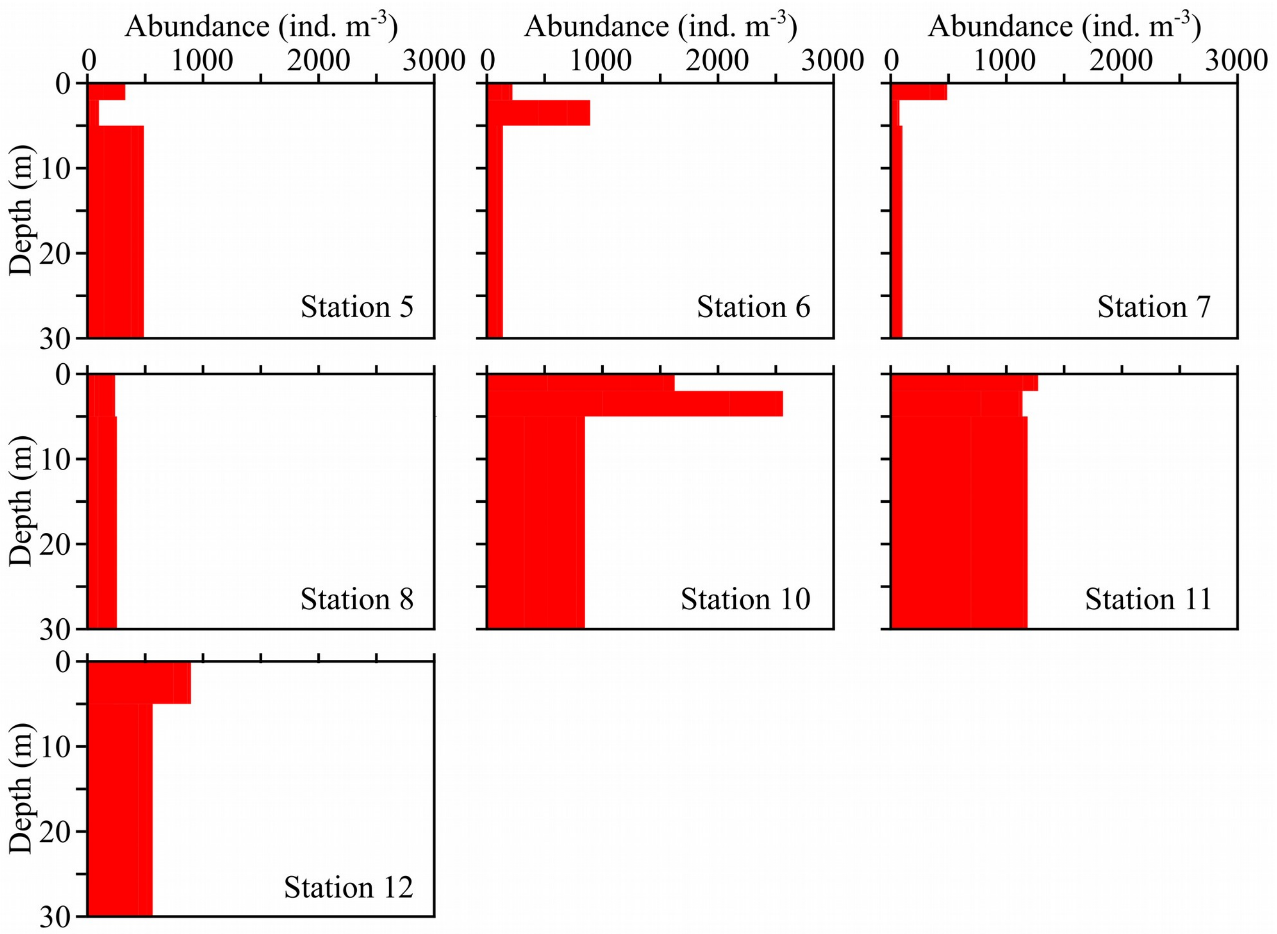

Fig. S3. Abundance and distribution of older developmental stages of *Calanus* spp. (copepodites IV and older) as sampled by vertical tows of a MultiNet in the upper 30 m at seven stations off the coast of northern Norway in May 2017. See Fig. 1 for the location of stations.
